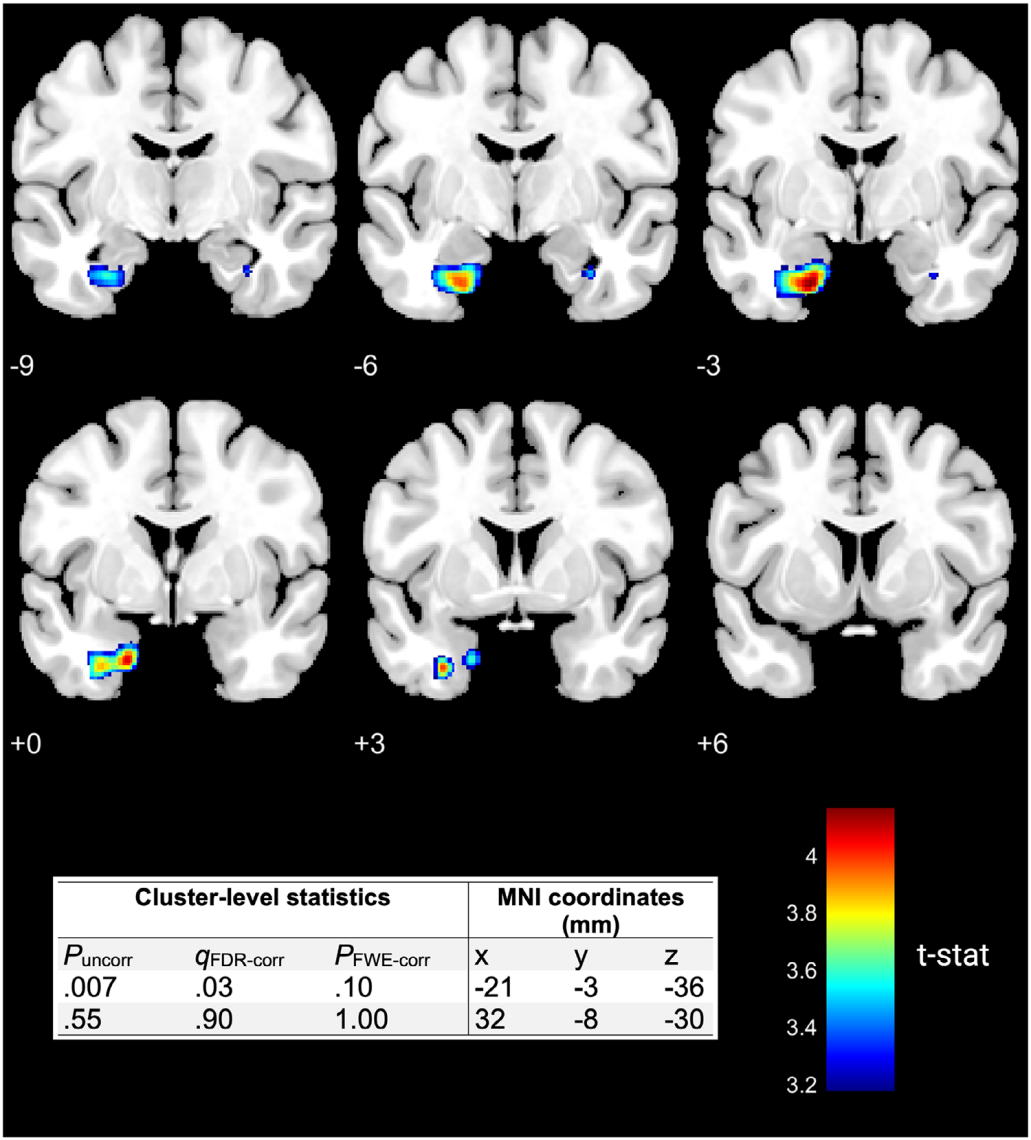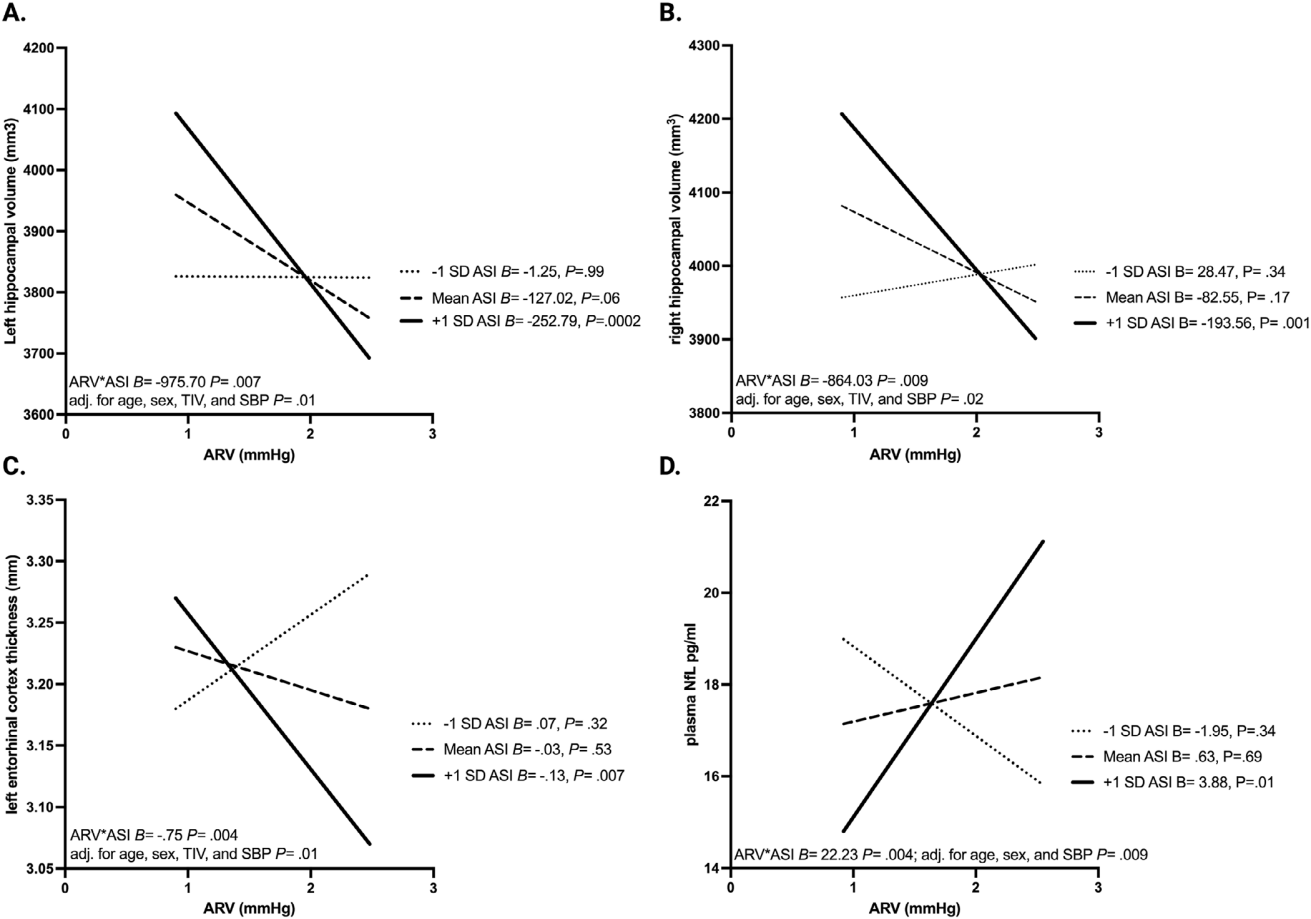# Blood Pressure Dynamic Instability and Neurodegeneration in Older Adults

**DOI:** 10.1002/alz70855_103424

**Published:** 2025-12-23

**Authors:** Trevor Lohman, Fatemah Shenasa, Isabel Sible, Arunima Kapoor, Allison C Engstrom, Shubir Dutt, Elizabeth Head, Lorena Sordo, John Paul M Alitin, Aimée Gaubert, Amy Nguyen, Neima Pahlevan, Daniel A. Nation

**Affiliations:** ^1^ Leonard Davis School of Gerontology, University of Southern California, Los Angeles, CA, USA; ^2^ University of California, Irvine, Irvine, CA, USA; ^3^ University of Southern California, Los Angeles, CA, USA; ^4^ Memory and Aging Center, UCSF Weill Institute for Neurosciences, University of California, San Francisco, San Francisco, CA, USA; ^5^ University of California Irvine, Irvine, CA, USA; ^6^ University of Southern California, Leonard Davis School of Gerontology, Los Angeles, CA, USA; ^7^ University of Southern Caliornia, Viterbi School of Medicine, Los Angeles, CA, USA

## Abstract

**Background:**

Blood pressure variability is an age‐related hemodynamic risk factor for neurodegenerative disease, but it remains unclear whether distinct forms of BPV display independent or interactive effects on brain health. Increased pulse pressure variability combined with high beat‐to‐beat average real variability may indicate greater hemodynamic instability, potentially exacerbating neurodegeneration.

**Method:**

Older adults (*N* = 105) underwent brain MRI and continuous BP monitoring to quantify beat‐to‐beat blood pressure variability using systolic average real variability (ARV) and pulse pressure variability through the ambulatory arterial stiffness index (AASI). The interactive effect of ARV and AASI on medial temporal lobe atrophy, plasma neurofilament light (NfL), and glial fibrillary acidic protein (GFAP) was studied.

**Result:**

The interaction between higher ARV and higher AASI was associated with decreased left (*p* = .01) and right (*p* = .02) hippocampal volumes and decreased left entorhinal cortex volume (*p* = .01) in a region of interest analysis adjusted for age, sex, total intracranial volume and average systolic blood pressure such that individuals with the highest ARV and AASI had the most adverse effects (*p* = .0002, *p* = .001, *p* = .007 respectively, Figure 1). This finding was confirmed with a voxel‐based morphometry analysis (Figure 2) which revealed left‐sided medial temporal lobe atrophy associated with the ARV*AASI interaction term (uncorrected *p* = .007, FDR‐corrected *p* = .03). The interactive effect was also significantly associated with increased plasma NfL (*p* = .009) such that individuals with the highest ARV and AASI had the highest NfL (*p* = .01) adjusted for age, sex, and average systolic blood pressure, but not GFAP.

**Conclusion:**

The interaction between higher ARV and higher AASI is independently associated with increased neurodegenerative markers in independently living older adults. Participants with the highest ARV and highest AASI displayed the most adverse markers of medial temporal lobe neurodegeneration.